# Machine learning-based infection prediction model for newly diagnosed multiple myeloma patients

**DOI:** 10.3389/fninf.2022.1063610

**Published:** 2023-01-13

**Authors:** Ting Peng, Leping Liu, Feiyang Liu, Liang Ding, Jing Liu, Han Zhou, Chong Liu

**Affiliations:** ^1^Department of Hematology, The Third Xiangya Hospital of Central South University, Changsha, China; ^2^Department of Pediatrics, The Third Xiangya Hospital of Central South University, Changsha, China

**Keywords:** infection, machine learning, multiple myeloma, prediction model, diagnosis

## Abstract

**Objective:**

To understand the infection characteristics and risk factors for infection by analyzing multicenter clinical data of newly diagnosed multiple myeloma (NDMM) patients.

**Methods:**

This study reviewed 564 NDMM patients from 2 large tertiary hospitals from January 2018 to December 2021, of whom 395 comprised the training set and 169 comprised the validation set. Thirty-eight variables from first admission records were collected, including patient demographic characteristics, clinical scores and characteristics, laboratory indicators, complications, and medication history, and key variables were screened using the Lasso method. Multiple machine learning algorithms were compared, and the best performing algorithm was used to build a machine learning prediction model. The model performance was evaluated using the AUC, accuracy, and Youden’s index. Finally, the SHAP package was used to assess two cases and demonstrate the application of the model.

**Results:**

In this study, 15 important key variables were selected, namely, age, ECOG, osteolytic disruption, VCD, neutrophils, lymphocytes, monocytes, hemoglobin, platelets, albumin, creatinine, lactate dehydrogenase, affected globulin, β2 microglobulin, and preventive medicine. The predictive performance of the XGBoost model was significantly better than that of the other models (AUROC: 0.8664), and it also performed well for the expected dataset (accuracy: 68.64%).

**Conclusion:**

A machine learning algorithm was used to establish an infection prediction model for NDMM patients that was simple, convenient, validated, and performed well in reducing the incidence of infection and improving the prognosis of patients.

## Background and purpose

Multiple myeloma (MM) is a malignant disease characterized by abnormal proliferation of clonal plasma cells; it accounts for 13% of hematological malignancies and is the second most common malignant tumor in the blood system. Active treatment methods for myeloma prolong the lifespan of myeloma patients, but the disease is incurable (Holmstrom et al., 2015; Valković et al., 2015). Infection is a common complication in patients with MM and is also the main cause of death. The number of deaths associated with infection in MM patients worldwide exceeds 80,000 annually, accounting for 2% of deaths from malignancies (Rajkumar, 2016). In a large UK study (Augustson et al., 2015), 45% of deaths within the first 2 months of treatment were due to infection. Infections account for 17% of all MM deaths and are a common cause of death among patients of all ages throughout the course of the disease. Therefore, it is necessary to study the infection status of patients with MM.

The mechanism of infection in patients with MM is relatively complex. In addition to the immunodeficiency caused by the disease itself (Faiman et al., 2018), in recent years, with the emergence of new treatment methods for MM, such as immunomodulators, proteasome inhibitors, monoclonal antibodies, and autologous stem cell transplantation, the survival of MM patients has improved, the risk of infection has increased, and the characteristics and spectrum of infection have changed (Tete et al., 2014; Blimark et al., 2015; Joshua et al., 2016; Park et al., 2017; Girmenia et al., 2019). In a population-controlled study in Sweden (Blimark et al., 2015), the risk of bacterial infection among patients with MM was 7 times that of the control group, and the overall risk of viral infection was 10 times higher. In terms of etiology, among 281 microbiologically defined infections (MDIs) studied by Teh et al., 152 were bacterial infections. There were 72 (47 4%), 59 (38 8%), and 21 (13 8%) infections caused by gram-negative (GN), gram-positive (GP), and multiple organisms, respectively. *Escherichia coli* was the most common isolate (23 7%), followed by *Clostridium difficile* (11 8%). In terms of infection time, previous studies have noted that infection is more common during the initial diagnosis and induction therapy (Lin et al., 2020). However, due to inconsistencies in the economic level and local epidemiology of each country, the infection situation of MM patients is also inconsistent. At present, the amount of research data related to infection in NDMM patients in China is relatively small, and the clinical data are not perfect. The use of antibiotics to prevent infection in MM patients with a high risk of infection based on a prediction model constructed with a single risk factor is controversial (Vesole et al., 2012; Drayson et al., 2019). If a prediction model for identifying patients with a high risk of infection is constructed based on complete clinical data and a large number of cases, this problem can be solved. Most of the current research on infection prediction models for patients with MM is based on traditional statistical methods. Shang et al. (2022) analyzed data from 914 patients at two centers and identified elevated ECOG scores, hemoglobin (anemia), B2 microglobulin, and GLB as factors associated with early infection and developed an IRMM model to classify patients into high-, intermediate-, and low-risk groups. A study by Dumontet et al. (2018) developed a predictive model for first TE ≥ grade 3 infection within the first 4 months of treatment in the Eastern Cooperative Oncology Group based on a multifactorial logistic regression analysis of data from 1,378 patients based on serum β2-microglobulin, lactate dehydrogenase, and hemoglobin levels to define high- and low-risk groups. Valkovic et al. collected retrospective data from 240 MM inpatients to create a numerical multiple myeloma infection risk index (MMIRI) to predict infections in myeloma patients. The results of the study showed that factors affecting the pathogenesis and incidence of infection included sex, physical status, Durie–Salmon disease stage, international staging system, serum creatinine level, immune paralysis, neutropenia, serum ferritin level, presence of any catheter, duration of disease, stable/progressive disease, and type of treatment (Valkovic et al., 2018). Nevertheless, no studies have used machine learning to build a model to predict the risk of infection in NDMM patients. Machine learning is an application of artificial intelligence that learns from data based on computational modeling. The advantage of machine learning is that it can be directly applied to individuals. For complex medical problems, such as processing and analyzing medical big data, the performance of machine learning is better than that of traditional methods. Statistical analysis is better, and performance on specific tasks improves with experience (Beam and Kohane, 2018).

This study analyzed the clinical data of 564 NDMM patients from multiple centers, revealed the characteristics of infection in MM patients, identified risk factors for infection, and used machine learning to build a model to predict the risk of infection in MM patients, which is helpful information when determining the use of antibiotics for infection prevention and treatment. The timing of other anti-infection measures and the early implementation of infection prevention strategies can reduce the incidence of infection and improve the prognosis of patients.

## Materials and methods

### Study subjects

The clinical data of 564 NDMM patients (349 males and 215 females) from January 2018 to December 2021 were collected through a medical record system.

### Inclusion and exclusion criteria

The inclusion criteria were (1) diagnoses that met the diagnostic criteria of the National Comprehensive Cancer Network (NCCN) and the International Myeloma Working Group (IMWG) (Rajkumar et al., 2014) and (2) complete medical records. The exclusion criteria were as follows: (1) patients with non-NDMM; (2) patients with psychiatric disorders or confusion and patients who could not cooperate; (3) patients who were transferred to the hospital for other reasons during the treatment period; and (4) patients with other infectious diseases or other malignant tumors.

### Study design and data collection

For all included patients, we obtained information on patient demographic characteristics (age, sex), clinical scores and characteristics (ECOG score, CVC, ureter, staging, DS, ISS, RISS), comorbidities (diabetes, tuberculosis, hepatitis, COPD, cardiovascular disease, chronic gastrointestinal disease, osteolytic destruction, extramedullary infiltration), medication history (chemotherapy regimens, infection prevention medication), and laboratory indicators [levels of neutrophils, lymphocytes, monocytes, eosinophils, basophils, hemoglobin, platelets, albumin, serum calcium, lactate dehydrogenase (LHD), affected globulin, and β2 microglobulin (β2MG)]. Patients who were already infected on admission and those with missing data were excluded.

### Data collection

All variables were obtained from the electronic medical record systems of both hospitals. Data included variables such as patient demographic characteristics, clinical scores and clinical features, comorbidities, medication history, and laboratory indicators. In total, 38 variables were collected for the first admission record. The Lasso method was also used to screen out key variables. Data entry was performed by physicians or medical students who were involved in this study.

### Definitions

Infections were defined as MDIs, clinically defined infections (CDIs), and fever of unknown origin.

Microbiologically defined infections were infections with a pathogen identified by microbiological testing of blood or secretion samples from any site. CDIs were observed when there was imaging evidence and clinical symptoms of infection after negative microbiological test results.

### Feature selection and data transformation

Only information from the first admission to the hospital before treatment was included in the model development, and patients were divided into infected and non-infected groups according to whether they were infected or not. Data units included from different hospitals were converted and harmonized; for example, a creatinine value of 1 mg/dL equaled 88.4 μmol/L. Medication-related variables were converted into ordinal variables as follows: 1 = VCD, 2 = VRD, 3 = VCD + VRD, 4 = CD38, and 5 = other chemotherapy regimens. The key variables were selected for subsequent modeling with the LassoCV approach.

First, a variety of machine learning algorithms were used to classify the data. These algorithms included the XGBoost, logistic regression, LightGBM, random forest, AdaBoost, and GaussianNB algorithms. The resampling method was used for verification. The samples were repeated 5 times, the validation set of each resampling training accounted for 30.000% of the total sample, and the training set accounted for 70.000%. This was to ensure that the selected training samples during training of multiple models were consistent and to better compare multiple models. The individual models were evaluated using the AUC, calibration plot, accuracy, sensitivity, specificity, positive predictive value, negative predictive value, F1 score, and Kappa value.

The best algorithm was selected by multimodel comparison and then remodeled using the best algorithm. The model parameters were as follows: the objective (optimization objective function) was binary logistic regression; the learning rate was 0.1; the maximum tree depth was 3; the Minimum Bifurcation Weights Sum was 9; and the regularization lambda was 3. Unlike the method based on a multimodel comparison, when modeling with the best performing algorithm, we randomly selected 10% of the overall sample as the test set, and the remaining samples were used as the training set for 5-fold cross-validation.

### Interpretation of the model

The Shapley additive explanations (SHAP) package (Python) interprets the output of a machine learning model, treating all features as “contributors,” and for each predicted sample, the model produces a predicted value. Its greatest advantage is that it can reflect the influence of the features in each sample and show the positive and negative influences. This study used the SHAP package to interpret the model. A SHAP value plot was used to show the contribution of each variable in the model. Model variable importance plots were used to show the importance rank of each variable. The force plot was used to exemplify how each variable affected the predicted outcome for each sample.

### Statistical analysis

This study used Python version 3.7, and the statsmodels 0.11.1 package in Python was used to determine whether the differences in each variable were statistically significant in the two populations. The analysis method was selected according to the distribution of the samples, the homogeneity of variance and the sample size. The chi-square test was used for categorical variables, and the Mann–Whitney *U* test was used for quantitative variables.

In this study, LassoCV was used to screen key variables using a 5-fold cross-validation method to automatically eliminate factors with coefficients of zero (sklearn 0.22.1 package in Python). Lasso resulted in a more refined model constructed with a penalty function; thus, some regression coefficients were compressed, i.e., the sum of the absolute values of the coefficients was forced to be less than some fixed value, and some regression coefficients were set to zero. Thus, retaining the advantage of subset shrinkage, it was a biased estimator dealing with data with complex covariance. In the multimodel and best model modeling process, the xgboost 1.2.1 package in Python was used for the XGBoost algorithm, the lightgbm 3.2.1 package in Python was used for the LightGBM algorithm, and the sklearn 0.22.1 package in Python was used for the other algorithms.

The SHAP 0.39.0 package in Python was used to demonstrate the interpretability of the model.

## Results

**FIGURE 1:**
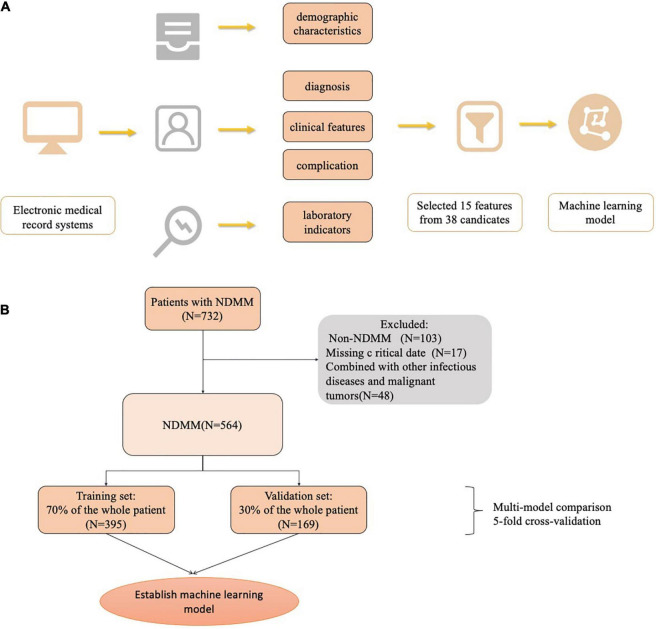
The workflow diagram of this study.

There were 564 patients in this study. During the multimodel comparison, 395 patients were included in the training set, and 169 patients were included in the validation set. Table 1 shows the baseline characteristics of the total population. The median age was 61.0 years (range 54.0–66.0). The IgG subtype (47.3%) accounted for the largest proportion of the population, followed by the IgA (25.1%), λ light chain (10.8%), κ light chain (8.7%), IgD (5.3%), double clone (1.96%), non-secretory (0.35%), and IgM (0.17%) subtypes. In the population, 249 (44.15%) patients were infected, and 315 (55.85%) patients were not infected. Among the infected patients, the lungs and upper respiratory tract were the most common infection sites in 81.1% of the patients, the urinary tract in 6.8%, and the gastrointestinal tract in 4%; a bloodstream infection and unexplained fever were observed in 1.1% of the patients. Figure 1 shows the flowchart of our research.

**TABLE 1:** Preoperative information.

Variable	All (*n* = 564)	Non-infection group (*n* = 315)	Infection group (*n* = 249)	*P*-value
Infection prevention medication, n (%)	347 (61.525)	201 (63.810)	146 (58.635)	0.210
	217 (38.475)	114 (36.190)	103 (41.365)	
VCD regimens, n (%)	233 (41.312)	117 (37.143)	116 (46.586)	0.024
	331 (58.688)	198 (62.857)	133 (53.414)	
VRD regimens, n (%)	551 (97.695)	310 (98.413)	241 (96.787)	0.201
	13 (2.305)	5 (1.587)	8 (3.213)	
VCD + VRD regimens, n (%)	428 (75.887)	246 (78.095)	182 (73.092)	0.168
	136 (24.113)	69 (21.905)	67 (26.908)	
CD38 regimens, n (%)	554 (98.227)	309 (98.095)	245 (98.394)	0.790
	10 (1.773)	*6 (1.905)*	*4 (1.606)*	
Other chemotherapy regimens, n (%)	553 (98.050)	311 (98.730)	242 (97.189)	0.189
	11 (1.950)	4 (1.270)	7 (2.811)	
Osteolytic destruction, n (%)	223 (39.539)	134 (42.540)	89 (35.743)	0.101
	341 (60.461)	181 (57.460)	160 (64.257)	
Extramedullary infiltration, n (%)	543 (96.277)	305 (96.825)	238 (95.582)	0.439
	21 (3.723)	10 (3.175)	11 (4.418)	
Hepatitis, n (%)	514 (91.135)	285 (90.476)	229 (91.968)	0.536
	50 (8.865)	30 (9.524)	20 (8.032)	
COPD, n (%)	504 (89.362)	285 (90.476)	219 (87.952)	0.334
	60 (10.638)	30 (9.524)	30 (12.048)	
Cardiovascular disease, n (%)	375 (66.489)	221 (70.159)	154 (61.847)	0.038
	189 (33.511)	94 (29.841)	95 (38.153)	
Chronic gastrointestinal disease, n (%)	485 (85.993)	271 (86.032)	214 (85.944)	0.976
	79 (14.007)	44 (13.968)	35 (14.056)	
Tuberculosis, n (%)	537 (95.213)	302 (95.873)	235 (94.378)	0.409
	27 (4.787)	13 (4.127)	14 (5.622)	
Diabetes, n (%)	505 (89.539)	281 (89.206)	224 (89.960)	0.772
	59 (10.461)	34 (10.794)	25 (10.040)	
RISS, n (%)	40 (7.092)	26 (8.254)	14 (5.622)	0.182
	338 (59.929)	194 (61.587)	144 (57.831)	
	186 (32.979)	95 (30.159)	91 (36.546)	
ISS, n (%)	85 (15.071)	59 (18.730)	26 (10.442)	0.003
	180 (31.915)	107 (33.968)	73 (29.317)	
	299 (53.014)	149 (47.302)	150 (60.241)	
DS, n (%)	16 (2.837)	12 (3.810)	4 (1.606)	0.090
	52 (9.220)	34 (10.794)	18 (7.229)	
	496 (87.943)	269 (85.397)	227 (91.165)	
Disease classification, n (%)	267 (47.340)	147 (46.667)	120 (48.193)	0.283
	142 (25.177)	83 (26.349)	59 (23.695)	
	110 (19.504)	60 (19.048)	50 (20.080)	
	30 (5.319)	20 (6.349)	10 (4.016)	
	15 (2.660)	5 (1.587)	10 (4.016)	
Ureter, staging, n (%)	540 (95.745)	306 (97.143)	234 (93.976)	0.064
	24 (4.255)	9 (2.857)	15 (6.024)	
CVC, n (%)	432 (76.596)	245 (77.778)	187 (75.100)	0.456
	132 (23.404)	70 (22.222)	62 (24.900)	
Sex, n (%)	349 (61.879)	194 (61.587)	155 (62.249)	0.872
	215 (38.121)	121 (38.413)	94 (37.751)	
B2GM, median [IQR]	5.530 [3.300, 10.610]	4.540 [3.100, 8.800]	6.360 [3.520, 12.110]	<0.001
Affected globulin, median [IQR]	25.500 [7.790, 51.600]	24.150 [7.010, 50.190]	29.000 [8.420,54.250]	0.189
LHD, median [IQR]	185.000 [148.000, 232.000]	180.000 [146.000, 225.000]	191.000 [149.000,238.000]	0.053
Creatinine, median [IQR]	94.000 [73.000, 180.800]	91.000 [70.200, 158.000]	102.000 [76.000,223.000]	0.012
Serum calcium, median [IQR]	2.280 [2.120, 2.500]	2.290 [2.130, 2.500]	2.270 [2.100,2.500]	0.266
Albumin, mean (±SD)	32.183 (±7.322)	33.189 (±7.471)	30.910 (±6.923)	<0.001
Platelets 10^9^/L, median [IQR]	161.000 [111.000, 218.000]	172.000 [122.000, 218.000]	148.000 [98.000, 219.000]	0.010
Hemoglobin g/L, median [IQR]	86.000 [69.000, 105.000]	89.000 [71.000, 108.000]	82.000 [69.000, 98.000]	0.010
Basophils, median [IQR]	0.010 [0.000, 0.020]	0.010 [0.000, 0.030]	0.010 [0.000, 0.020]	0.016
Eosinophils, median [IQR]	0.060 [0.000, 0.110]	0.080 [0.030, 0.110]	0.030 [0.000, 0.100]	<0.001
Monocytes, median [IQR]	0.400 [0.300, 0.590]	0.400 [0.300, 0.500]	0.460 [0.300, 0.700]	0.002
Lymphocyte 10^12^/L, median [IQR]	1.200 [0.800, 1.660]	1.300 [1.000,1.720]	0.940[0.500, 1.470]	<0.001
Neutrophil 10^9^/L, median [IQR]	3.400 [2.300, 5.620]	3.100 [2.300,4.000]	5.120[2.000, 8.610]	<0.001
ECOG score, median [IQR]	2.000 [1.000, 3.000]	2.000 [1.000,3.000]	2.000[2.000, 3.000]	<0.001
Age, median [IQR]	61.000 [54.000, 66.000]	60.000 [53.000,66.000]	62.000[55.000, 67.000]	0.086

VCD, Bortezomib, cyclophosphamide, dexamethasone; VRD, Bortezomib, Lenalidomide, dexamethasone; COPD, Chronic obstructive pulmonary Disease; ISS, International Staging System; DS, Durie-Salmon; CVC, Central venous Catheter; ECOG, Eastern Cooperative Oncology Group; LHD, Lactate dehydrogenase.

### Variable filter

A total of 15 key factors were selected by the LassoCV method: “age,” “ECOG score,” “osteolytic destruction,” “VCD,” “neutrophil count,” “lymphocyte count,” “monocytes,” “hemoglobin,” “platelet,” “albumin,” “creatinine,” “lactate dehydrogenase,” “affected globulin,” “B2 microglobulin,” and “infection prevention medication.”

### Multialgorithm model comparison

**FIGURE 2:**
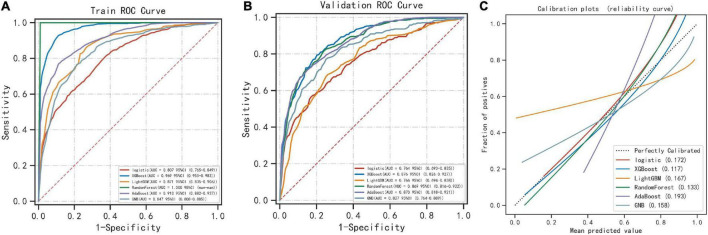
Comparison of 6 machine learning algorithms. **(A)** The ROC results of the models established by 6 machine learning algorithms in the training set. **(B)** The ROC results of the models established by 6 machine learning algorithms in the validation set. **(C)** Calibration plots of models built by 6 machine learning algorithms.

Six machine learning models were used to classify the sample data. Among the six different machine learning algorithms, XGBoost performed the best, with AUCs of 0.969 and 0.876 in the training and validation sets, respectively (Figures 2A, B). It also performed the best in the calibration curve graph (Figure 2C). Additionally, its cutoff value, accuracy, sensitivity, specificity, positive predictive value, negative predictive value, F1 score, and Kappa value in the training set were 0.452, 0.911, 0.921, 0.908, 0.888, 0.931, 0.904, and 0.820, respectively. The indices of the other machine learning algorithms are shown in Table 2 and Supplementary Table 1.

**TABLE 2:** Multi-model classification–training set results.

Model	AUC (SD)	Cut off (SD)	Accuracy (SD)	Sensitivity (SD)	Specificity (SD)	Positive predictive value (SD)	Negative predictive value (SD)	F1 score (SD)	Kappa (SD)
Logistic	0.807 (0.024)	0.428 (0.098)	0.730 (0.010)	0.724 (0.147)	0.741 (0.114)	0.704 (0.064)	0.782 (0.067)	0.698 (0.047)	0.455 (0.028)
XGBoost	0.969 (0.004)	0.452 (0.029)	0.911 (0.005)	0.921 (0.014)	0.908 (0.015)	0.888 (0.019)	0.931 (0.011)	0.904 (0.007)	0.820 (0.011)
LightGBM	0.871 (0.033)	0.418 (0.126)	0.812 (0.012)	0.832 (0.063)	0.810 (0.070)	0.796 (0.064)	0.840 (0.044)	0.808 (0.010)	0.619 (0.019)
RandomForest	1.000 (0.000)	0.620 (0.040)	0.996 (0.002)	1.000 (0.000)	1.000 (0.000)	1.000 (0.000)	0.994 (0.003)	1.000 (0.000)	0.993 (0.004)
AdaBoost	0.910 (0.007)	0.482 (0.005)	0.834 (0.008)	0.776 (0.016)	0.884 (0.011)	0.847 (0.005)	0.825 (0.012)	0.810 (0.009)	0.659 (0.017)
GNB	0.847 (0.007)	0.284 (0.094)	0.783 (0.012)	0.800 (0.064)	0.774 (0.062)	0.741 (0.040)	0.829 (0.036)	0.766 (0.011)	0.563 (0.021)

### Best algorithm model

**FIGURE 3:**
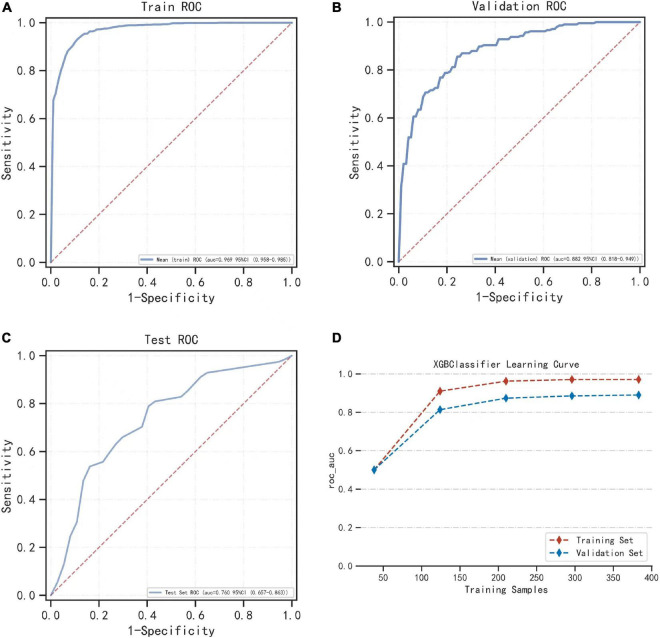
The performance of the model built by the XGBoost algorithm. **(A)** The ROC result of the model established by the XGBoost algorithm in the training set. **(B)** The ROC result of the model established by the XGBoost algorithm in the validation set. **(C)** Shows the results of the ROC of the model established by the XGBoost algorithm in the training set and the verification set according to the change of the sample size. **(D)** During cross-validation, the ROC of the training set and the validation set varies with the sample size of the training set.

After comparing multiple models, the XGBoost model performed the best, and we used XGBoost for modeling analysis. We randomly selected 10% of the total sample as the test set, and the remaining samples were used as the training set for 5-fold cross-validation. The AUC of the XGBoost model was 0.971 in the training set, 0.884 in the validation set, and 0.760 in the test set (Figures 3A–C). Additionally, during cross-validation, when the training samples reached 200, the AUC of the model reached as table state (Figure 3D). Supplementary Tables 2–4 show the model evaluation metrics for the training set, validation set, and test set, respectively.

### Model interpretability

**FIGURE 4:**
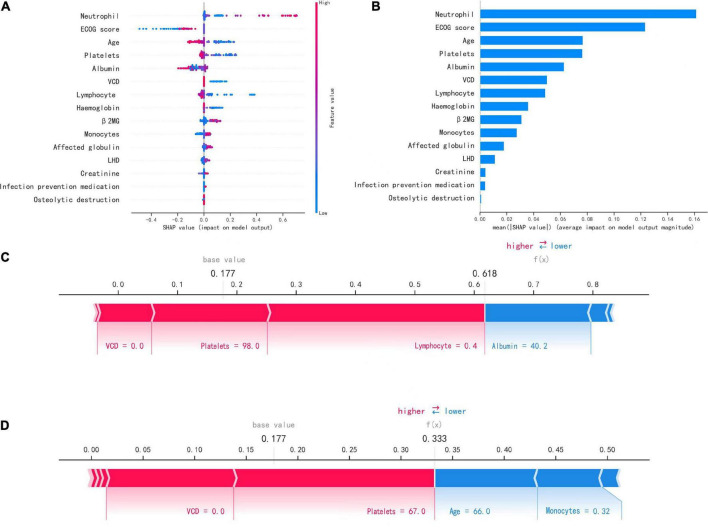
Interpretation of the model. **(A)** SHAP plot of 15 key variables. **(B)** Importance ranking chart of 15 key variables. **(C,D)** Show patients with positive (infected) and negative (uninfected) predictions, respectively.

The SHAP plot in Figure 4A shows how each variable in the validation set contributed to predicting infection. The redder each point is, the larger the absolute value of the point, and the bluer the point is, the smaller the absolute value of the point. The larger the absolute value of the negative ordinate is, the greater the possibility of the predicted result being negative, and the greater the absolute value of the positive ordinate is, the greater the possibility of the predicted result being positive.

For example, the larger the neutrophil count is, the more likely the patient is to have an infection, and the higher the platelet count is, the less likely the patient is to have an infection. Figure 4B shows the importance ranking of each variable. Neutrophil count, ECOG score, and age were the most important variables.

Figures 4C, D show how the variables of the two samples affected the results with two force plots. As shown in Figure 4C, the model predicted a positive outcome for the patient who actually developed an infection. The longest segment in the red part in the figure was the lymphocyte count (0.4*10^9^/L), indicating that the lymphocyte count had the largest positive contribution to the outcome of infection in this patient, and the second largest positive impact on the outcome was the platelet count (98*10^9^/L). In Figure 4D, the model predicted a negative outcome for the patient who was actually uninfected. The two variables with the most positive effects were the platelet count (67*10^9^/L) and VCD (0.0), and the variables with the most negative effects on the outcome were the age and monocyte count (66.0 and 0.32*10^9^/L).

## Discussion

Patients with MM have varying degrees of immunodeficiency, which increases the risk of serious infection, and this study found that infection is common among patients with MM and appears to be the main cause of initial presentation and poor prognosis. This study reviewed 564 patients with NDMM in two large tertiary hospitals from January 2018 to December 2021, analyzed the infection of NDMM patients, and provided insights into the assessment, prevention and treatment of MM patients. In all, 395 cases comprised the training set, and 169 cases comprised the validation set. Thirty-eight variables recorded on the first admission were collected, including patient demographics, clinical scores and characteristics, laboratory indicators, complications, and drug history. Key variables were screened out using the Lasso method. Multiple machine learning algorithms were compared, and the best performing algorithm was used to establish a machine learning prediction model. The AUC, accuracy, Youden’s index and other indicators were used to evaluate the model performance. Finally, the SHAP package was used to demonstrate the application of the model in two cases.

In this study, the infection rate of NDMM patients on initial admission was as high as 44.15%; 152 of the 328 MM patients in the study by Song Bin et al. had nosocomial infection, and the infection rate was 46.3%, which was similar to the results of this study. Valkovic et al. found an infection incidence of 17.9% (43/240) in their study, which was somewhat different from the results of this study. There may be a certain relationship between hospitals in China and abroad and the different modes of infection control and clinical management of MM patients; hospitals in foreign countries emphasize early detection, early diagnosis, and early treatment of MM, which may be the reason why the incidence of infection obtained in foreign studies is lower than that in China. The most common sites of infection were the lungs and upper respiratory tract, which were similar to results from previous studies (Dumontet et al., 2018; Faiman et al., 2018; Lin et al., 2020; Shang et al., 2022). These were followed by infections of the urinary tract, gastrointestinal tract, and bloodstream.

This study analyzed 564 NDMM patients at the time of initial admission and used machine learning to establish an infection prediction model that contained 15 important key variables, including age, ECOG score, osteolytic destruction, VCD, neutrophil count, lymphocyte count, monocyte count, hemoglobin, platelets, albumin, creatinine, lactate dehydrogenase, affected globulin, and B2 microglobulin. A retrospective study by Valkovic et al. analyzed the clinical data of 240 MM inpatients and found that the susceptibility factors for the development of infection in MM patients were female sex (*p* = 0.001), poor general condition (*p* < 0.001), DS stage III group B (advanced) disease duration (*p* = 0.007), elevated serum creatinine level (*p* = 0.036), neutropenia (*p* = 0.009), indwelling catheterization (*p* < 0.001), granulocytopenia (*p* = 0.009), and elevated serum ferritin level (*p* = 0.001) (Valković et al., 2015). Sørrig et al. (2019) retrospectively analyzed all infectious complications in 2,557 patients within 6 months after diagnosis of MM through the Danish registry, showing that pulmonary infections and bloodstream infections accounted for 46% of total infections and that multivariate analysis indicated that risk factors for pulmonary infections in MM patients were male sex (*p* = 0.001), ISS stage II (*p* = 0.0004) and III (*p* = 0.0004), and elevated lactate dehydrogenase level (*p* = 0.0008). The key factors included in this study have been reported in previous studies, including ECOG score, hemoglobin, B2 microglobulin, affected globulin, lactate dehydrogenase, serum creatinine, and neutropenia, and 8 other risk factors associated with the development of infection were included in this study’s prediction model, which helps to more fully predict the risk of infection among patients with newly diagnosed myeloma. In recent years, studies have found that platelets play an important role in initiating the inflammatory response and immune regulation, and at present, platelet concentrates such as platelet-rich plasma (PRP) have achieved significant clinical efficacy in the treatment of chronic wounds (Yuan et al., 2009; Miron et al., 2017). The mechanism is not only the release of growth factors after platelet activation but also the anti-infection effect of platelets, which is one of the reasons they promote wound healing. Lymphopenia has been shown to indicate the presence of immunosuppressive states (Teh et al., 2016; Ying et al., 2017), and patients with lymphopenia have an inadequate immune response and are susceptible to bacterial infections, consistent with a positive effect on lymphocyte counts in the model. Bortezomib disrupts intracellular proteasome function and NF-kB activation (Teh et al., 2015; Li and Wang, 2019), leading to selective depletion of T cells and decreased viral antigen presentation, increasing the risk of viral reactivation associated with the use of bortezomib zoster virus reactivation (up to 36%). Therefore, prophylaxis with acyclovir or valacyclovir during treatment with PI is now commonly recommended (Eisen et al., 2003; Teh et al., 2014). In addition, seven factors, including albumin, osteolytic destruction, age, and creatinine, are extremely important. When the level of albumin in the blood is low, the body’s immunity is low, and the chance of infection increases. When bone pain symptoms occur during osteolytic destruction, normal lung ventilation and lung ventilation function are affected, and pathogens easily invade the respiratory system and are stored in the lungs and difficult to eliminate. Regardless of the presence of MM, older patients are particularly susceptible to infection, with a higher morbidity and at least a three times higher mortality than younger patients (Yoshikawa, 2000; Nucci and Anaissie, 2009; Solana et al., 2012), which may be related to age-related immune dysfunction. When the patient’s creatinine is elevated and renal insufficiency occurs, the body’s immune system is easily damaged due to the accumulation of toxins in the body and acidosis. Therefore, the above factors can be included in the infection risk model.

In this study, the model was evaluated on its ability to predict infection in NDMM patients, and nine machine learning models were used to classify the data sample. XGBoost performed best among the nine different machine learning algorithms, with AUCs of 0.971 and 0.884 in the training and validation sets, respectively, and an AUC = 0.760 in the final model in the test set (Figure 3). Additionally, when cross-validation was performed, the model reached a stable state when the sample size of the training and validation sets reached 200. Thus, we developed an infection prediction model for NDMM patients with great predictive power. This was a multicenter study, which was also an advantage over other studies; rich data allow for rigorous evaluation of the performance of machine learning models. Therefore, the model established in this study can be used to predict the risk of infection among patients with NDMM, help determine the timing for the use of antimicrobials and other anti-infective measures, implement early infection prevention strategies, reduce the incidence of infection, improve patient outcomes, and reduce the economic burden on patients by reducing the length of hospital stay and reducing hospitalization costs for most patients. In addition, machine learning can be applied to the diagnosis, prognosis, and treatment options for MM.

This study has several limitations. One is that, as with other retrospective studies, some patients were excluded due to missing key data, resulting in selection bias; nevertheless, participants from multiple hospitals were assessed and indicators that were readily available for routine testing were evaluated. Second, our model can be used to predict the risk of developing infection, but the risk of a specific type of infection cannot be clearly classified; however, clinicians can decide to take appropriate precautions based on clinical experience. For example, when using bortezomib regimens, antiviral drugs such as valacyclovir can be used prophylactically to prevent herpes zoster virus activation, as bortezomib leads to selective depletion of T cells and decreased viral antigen presentation, leading to an increased risk of viral reactivation. Finally, while SHAP values were used to help explain our machine learning models, there is still a need for a more interpretable model in clinical practice (Rudin, 2019). In future work, we plan to develop automated clinical scoring systems based on nomograms or machine learning based on our data to provide clinicians with more practical and easy-to-understand tools (Xie et al., 2020).

## Conclusion

In conclusion, this study found that infections are common among NDMM patients. The XGBoost machine learning algorithm was used to build an infection prediction model for NDMM patients with easy operation and good performance with an AUC of 0.884. This model can help determine the timing of the preventive use of antibiotics and other anti-infection measures and has important clinical significance for early implementation of infection prevention strategies to improve patient outcomes.

## Data availability statement

The original contributions presented in this study are included in this article/Supplementary material, further inquiries can be directed to the corresponding author.

## Author contributions

TP, LL, and JL designed and performed the study. TP, LL, FL, LD, HZ, and CL collected the data. LL processed statistical data. TP drafted the manuscript under the guidance of JL. All authors contributed to the article and approved the submitted version.
