# Supplementary material for: Machine learning-based infection prediction model for newly diagnosed multiple myeloma patients
Source: Front Neuroinform. 2023 Jan 13;16:1063610. doi: 10.3389/fninf.2022.1063610 (PMC9880856; doi:10.3389/fninf.2022.1063610)
Supplement: Supplementary file 1 [file Table_1.docx]

Supplementary Table 1 Multi-model classification-validation set results summary:

| Model | | AUC(SD) | Cut off(SD) | Accuracy(SD) | Sensitivity(SD) | Specsitivity(SD) | Positive predictive value(SD) | negative predictive value(SD) | F1 Score(SD) | Kappa(SD) |
| --- | --- | --- | --- | --- | --- | --- | --- | --- | --- | --- |
| logistic | 0.764(0.062) | 0.428(0.098) | 0.699(0.057) | 0.622(0.169) | 0.804(0.114) | 0.694(0.106) | 0.731(0.070) | 0.638(0.109) | 0.382(0.108) |  |
| XGBoost | 0.876(0.030) | 0.452(0.029) | 0.794(0.029) | 0.853(0.052) | 0.762(0.070) | 0.764(0.033) | 0.819(0.031) | 0.805(0.032) | 0.581(0.056) |  |
| LightGBM | 0.766(0.092) | 0.418(0.126) | 0.721(0.057) | 0.833(0.062) | 0.658(0.098) | 0.690(0.070) | 0.758(0.064) | 0.754(0.066) | 0.435(0.110) |  |
| RandomForest | 0.869(0.026) | 0.620(0.040) | 0.761(0.017) | 0.803(0.044) | 0.801(0.065) | 0.852(0.061) | 0.728(0.037) | 0.825(0.032) | 0.497(0.025) |  |
| AdaBoost | 0.870(0.024) | 0.482(0.005) | 0.791(0.018) | 0.745(0.068) | 0.840(0.057) | 0.794(0.012) | 0.787(0.029) | 0.768(0.038) | 0.569(0.033) |  |
| GNB | 0.827(0.033) | 0.284(0.094) | 0.736(0.035) | 0.743(0.107) | 0.795(0.055) | 0.699(0.069) | 0.781(0.050) | 0.719(0.084) | 0.468(0.068) |  |
