# Supplementary material for: Machine learning-based infection prediction model for newly diagnosed multiple myeloma patients
Source: Front Neuroinform. 2023 Jan 13;16:1063610. doi: 10.3389/fninf.2022.1063610 (PMC9880856; doi:10.3389/fninf.2022.1063610)
Supplement: Supplementary file 2 [file Table_2.docx]

Supplementary Table 2 Training set results

| AUC(SD) | cutoff(SD) | Accuracy(SD) | Sensitivity(SD) | Specsitivity(SD) | Positive predictive value(SD) | negative predictive value(SD) | F1 Score(SD) |
| --- | --- | --- | --- | --- | --- | --- | --- |
| 0.971 (0.002) | 0.439 (0.023) | 0.913 (0.005) | 0.930 (0.016) | 0.904 (0.013) | 0.881 (0.013) | 0.940 (0.012) | 0.905 (0.006) |
