# Supplementary material for: Machine learning-based infection prediction model for newly diagnosed multiple myeloma patients
Source: Front Neuroinform. 2023 Jan 13;16:1063610. doi: 10.3389/fninf.2022.1063610 (PMC9880856; doi:10.3389/fninf.2022.1063610)
Supplement: Supplementary file 3 [file Table_3.docx]

Supplementary Table 3 Validation set results

| AUC(SD) | cutoff(SD) | Accuracy(SD) | Sensitivity(SD) | Specsitivity(SD) | Positive predictive value(SD) | negative predictive value(SD) | F1 Score(SD) |
| --- | --- | --- | --- | --- | --- | --- | --- |
| 0.884 (0.034) | 0.439 (0.023) | 0.785 (0.047) | 0.817 (0.101) | 0.823 (0.066) | 0.756 (0.061) | 0.810 (0.041) | 0.784 (0.073) |
