# Supplementary material for: Machine learning-based infection prediction model for newly diagnosed multiple myeloma patients
Source: Front Neuroinform. 2023 Jan 13;16:1063610. doi: 10.3389/fninf.2022.1063610 (PMC9880856; doi:10.3389/fninf.2022.1063610)
Supplement: Supplementary file 4 [file Table_4.docx]

Supplementary Table 4 Test set results

| AUC | cutoff | Accuracy(SD) | Sensitivity(SD) | Specsitivity(SD) | Positive predictive value(SD) | negative predictive value(SD) | F1 Score(SD) |
| --- | --- | --- | --- | --- | --- | --- | --- |
| 0.760 | 0.438 | 0.706 | 0.829 | 0.591 | 0.711 | 0.702 | 0.765 |
